# Methods to split cognitive task data for estimating split-half reliability: A comprehensive review and systematic assessment

**DOI:** 10.3758/s13423-021-01948-3

**Published:** 2021-06-07

**Authors:** Thomas Pronk, Dylan Molenaar, Reinout W. Wiers, Jaap Murre

**Affiliations:** 1 Faculty of Social and Behavioural Sciences, Department of Psychology, University of Amsterdam, P.O. Box: 15969, 1001 NL Amsterdam, The Netherlands; 2 Faculty of Social and Behavioural Sciences, Behavioural Science Lab, University of Amsterdam, Amsterdam, The Netherlands; 3 Open Science Tools (PsychoPy) Lab, School of Psychology, University of Nottingham, Nottingham, United Kingdom; 4 Faculty of Social and Behavioural Sciences, Department of Psychology, Addiction Development and Psychopathology (ADAPT) lab, University of Amsterdam, Amsterdam, The Netherlands; 5 Center for Urban Mental Health, University of Amsterdam, Amsterdam, The Netherlands

**Keywords:** Cognitive tasks, Confounding effects, Non-linear scoring algorithms, Reliability

## Abstract

Estimating the reliability of cognitive task datasets is commonly done via split-half methods. We review four methods that differ in how the trials are split into parts: a first-second half split, an odd-even trial split, a permutated split, and a Monte Carlo-based split. Additionally, each splitting method could be combined with stratification by task design. These methods are reviewed in terms of the degree to which they are confounded with four effects that may occur in cognitive tasks: effects of time, task design, trial sampling, and non-linear scoring. Based on the theoretical review, we recommend Monte Carlo splitting (possibly in combination with stratification by task design) as being the most robust method with respect to the four confounds considered. Next, we estimated the reliabilities of the main outcome variables from four cognitive task datasets, each (typically) scored with a different non-linear algorithm, by systematically applying each splitting method. Differences between methods were interpreted in terms of confounding effects inflating or attenuating reliability estimates. For three task datasets, our findings were consistent with our model of confounding effects. Evidence for confounding effects was strong for time and task design and weak for non-linear scoring. When confounding effects occurred, they attenuated reliability estimates. For one task dataset, findings were inconsistent with our model but they may offer indicators for assessing whether a split-half reliability estimate is appropriate. Additionally, we make suggestions on further research of reliability estimation, supported by a compendium R package that implements each of the splitting methods reviewed here.

## Introduction

Recently, it was suggested that a range of cognitive tasks may reliably measure group differences but not individual differences (Hedge et al., 2018). As cognitive tests are commonly used to make inferences about individuals, it seems a worthwhile pursuit to improve the reliability with which individual differences are assessed using these tests. To judge the success of such pursuits, accurate estimates of reliability are required. Reliability can be estimated via a range of coefficients, such as Cronbach’s alpha for questionnaire data. Traditionally, alpha is estimated by fitting a model of essential tau-equivalence on item-level data; each item has the same loading on the true score but has a unique mean and error variance (Cortina, 1993). The same approach may not translate well to cognitive tasks, due to their designs and scoring algorithms. For instance, task designs may contain random sequences of trials and repetitions of the same trial, while task-scoring algorithms may selectively exclude trials and apply non-linear transformations to trial scores. Instead, one could estimate reliability via a test-retest approach. However, administering the same task twice may not always be appropriate due to feasibility constraints or the task score varying over test-retest time frames, for example, due to learning effects.

An alternative approach that has been popular with cognitive tasks is split-half reliability. Trials are distributed across two parts, a score is calculated per part, and a two-part reliability coefficient, such as a Spearman-Brown adjusted Pearson correlation, is calculated between the two sets of part scores. Because split-half reliability is estimated via scores of aggregates of trials, practical issues with models for individual trials can be sidestepped. However, is the coefficient obtained an accurate estimate of reliability? One factor that may affect the accuracy of split-half reliability estimates is the method by which the task is split. A variety of methods have been proposed and applied to cognitive task data. To the best of our knowledge, these splitting methods have not yet been comprehensively reviewed nor systematically assessed, so we aimed to conduct such an examination.

In the remainder of the *Introduction*, we review four splitting methods, and a stratification approach, each having been used in the cognitive task literature. We evaluate each method on its ability to control for four possibly confounding effects. Based on our review, we make a theory-based recommendation as to which splitting method(s) may be considered most robust. In the empirical application, we estimated the reliabilities of four cognitive task datasets and associated scoring algorithms by systematically applying each splitting method. We compared estimates between splitting methods and tasks, to examine which confounding effects likely occurred.

### Confounds with time

The first effect we review is time: This effect can manifest, for example, when participants learn or become fatigued throughout a task. First-second halves splitting assigns trials to each part based on whether they belonged to the first or second half of the sequence administered to a participant. Since first-second splitting assigns early trials to one part and late trials to the other, first-second splitting is confounded with time effects. This confound has been used to argue against first-second splitting (Webb et al., 2006). Time effects have also been used to explain comparatively low reliability estimates found with first-second splitting of a Go/No-Go (GNG) task (Williams & Kaufmann, 2012), a Wisconsin Card Sorting Test (Kopp et al., 2021), and a comparable splitting method of a learning referent task (Green et al., 2016). Time effects can be controlled by balancing early and late trials between parts, which can be achieved by splitting trials based on whether their position in the sequence was odd or even.

### Confounds with task design

While odd-even splitting controls well for time, it can be confounded with task design. For instance, if a task features an alternating sequence of conditions, such as target and attribute trials in an Implicit Association Task (IAT) (Greenwald et al., 1998), an odd-even split would exclusively assign target trials to one part and attribute trials to the other. To demonstrate this effect, Green et al. (2016) stratified splits such that they were either confounded with task conditions (split by name) or balanced between task conditions (split by equivalence). It was shown that the split by equivalence yielded higher reliability estimates than the split by name. These relatively high estimates were accepted as most accurate. In contrast, comparatively high reliabilities of odd-even splits were found on scores of a Stop Signal Task (SST). These relatively high reliabilities were rejected as inaccurate for being an artifact of the task’s tracking procedure (Hedge et al., 2018). Hence, confounds between task design and odd-even splitting have been used to argue that odd-even splits can yield both overestimations and underestimations of reliability.

Both overestimation and underestimation of reliability can occur depending on how such confounds violate the measurement model assumed by a reliability coefficient. For instance, in a model of essential tau equivalence, correlated errors may inflate reliability while unequal loadings of trials on a true score may attenuate it (Green et al., 2016). Regardless, we assume that any splitting method that controls for task design yields a more accurate reliability estimate, and that confounds between task design and splitting method may yield either overestimations or underestimations in a given task dataset. We conceptualize controlling for task design by balancing conditions between parts as stratified splitting: strata are constructed from the trials that belong to each condition. Each stratum is then split into two parts by applying another splitting method. This approach ensures that strata are balanced between parts and allows direct comparison of splitting methods that are confounded with task design and spitting methods that are not confounded with task design.

### Confounds with trial sampling

Each of the splitting methods reviewed so far (first-second and odd-even in combination with stratification) constructs a single pair of parts for each participant. Regarding this pair as a sample, we will collectively refer to these methods as single-sample methods. Single-sample splits have been popular, perhaps in part for being relatively easy to perform. However, besides that they may be confounded with time and task design, they can also be subject to trial-sampling effects; any single split may yield anomalously high or low reliability estimates, purely by chance. In contrast, resampling methods estimate reliability by averaging multiple coefficients calculated from parts that are composed of randomly sampled trials. Some of these coefficients may yield overestimations of reliability and some underestimations. On the aggregate level, trial sampling effects are attenuated by averaging coefficients from each random sample of trials. Resampled splitting may benefit less from stratification than single-sample splitting since anomalously high or low reliability estimates that are an artifact from a confound between one particular split and the task design are already averaged out. However, stratification could still benefit resampled splitting by making splits more equivalent.

We distinguish two resampling methods, which differ in whether they construct parts by randomly sampling with or without replacement. The latter has been known variously as random sample of split-halves (Williams & Kaufmann, 2012), random splitting (Kopp et al., 2021), and bootstrapped splitting (Parsons et al., 2019). We use the term *permutated splitting* to emphasize that this method samples without replacement. Reliability coefficients calculated from permutated splits have a statistical quality that facilitates their interpretation: for coefficients such as the Spearman-Brown-adjusted Pearson correlation and two-part Cronbach’s alphas, if trial scores meet the measurement model assumptions, the mean coefficient of all possible splits approximates the test-retest correlation (Novick & Lewis, 1967; Warrens, 2015). In turn, this can be approximated by a large number of permutated splits. Parsons et al. (2019) recommended a minimum of 5,000 replications.

Averaging resampled coefficients may attenuate confounding effects, but their distributions may be useful to identify issues with a task dataset. Williams and Kaufmann (2012) suggested that wide and platykurtic resampled distributions may indicate trials that are poor quality, heterogeneous, or tapping multiple constructs. We have not formulated any guidelines nor hypotheses on the shapes of the distributions. This is in part because we are unsure how to interpret these distributions in terms of confounding effects. Additionally, we expect variation in width and skew of distributions as a function of reliability, because reliability coefficients have an upper bound of 1.

However, acknowledging that resampled distributions can be informative, we have made an explorative assessment of their range, skew, and kurtosis. Additionally, we explored whether reliability estimates of resampled coefficients varied as a function of stratification. We have used the distributions of permutated coefficients to qualify reliability estimates of single-sample splitting methods. The percentile of a reliability estimate of a single-sample splitting in the empirical cumulative distributions of non-stratified permutated coefficients indicates how extreme that estimate is compared to any random splitting method.

### Confounds with non-linear scoring

Continuing to sampling with replacement, we first note that each splitting method reviewed above constructs parts that are half of the length of the original task. Reliability coefficients estimate the reliability for the full-length task based on half-length scores and certain measurement model assumptions. An assumption shared by various reliability coefficients is a linear relation between task score and trial scores. Linearity is assumed by alpha (Green et al., 2016), Spearman-Brown-adjusted intraclass correlation coefficient (de Vet et al., 2017; Hedge et al., 2018; Warrens, 2017), and Spearman-Brown-adjusted Pearson correlation (Abacioglu et al., 2019; Chapman et al., 2019; Cooper et al., 2017; de Hullu et al., 2011; Enock et al., 2014; Lancee et al., 2017; MacLeod et al., 2010; Schmitz et al., 2019; Waechter et al., 2014). However, task-scoring algorithms may apply a range of non-linear transformations, thereby being incompatible with these coefficients by design.

Williams and Kaufmann (2012) proposed a splitting procedure that could be more robust with non-linear-scoring algorithms. This procedure constructs two parts of the same length of the original task by randomly sampling with replacement. As the two parts are full-length tasks, reliability can be estimated via a test-retest approach by correlating scores of these parts. Williams and Kaufmann (2012) referred to this method as a *Monte Carlo alpha-like coefficient,* which we will abbreviate to Monte Carlo splitting. This splitting method makes relatively weak assumptions about the relationship between trial and task scores, such as linearity. Hence, it could provide a more accurate reliability estimate of scores calculated via non-linear algorithms. Regardless, in a Go/No-Go (GNG) dataset scored via d’, Pearson correlations calculated from Monte Carlo splits yielded similar reliability estimates to Spearman-Brown-adjusted Pearson correlations calculated from permutated splits (Williams & Kaufmann, 2012).

### Interim summary

In summary, we reviewed four effects that may confound splitting methods and so affect the accuracy of split-half reliability estimates: time, task design, trial sampling, and non-linear scoring. We listed two splitting methods that produce a single sample of parts based on trial sequence (first-second and odd-even) and two that randomly resample parts (permutated and Monte Carlo). A fifth method, stratified splitting, balances conditions between parts by constructing strata that are next split with one of the other methods. We have argued that confounding effects are likely to interact with splitting methods as follows: Time is confounded with first-second and controlled for by odd-even. Task design can be confounded with single-sample splitting and controlled for by stratification. Resampled splitting controls for confounds by averaging them out. Non-linear scoring is confounded with first-second, odd-even, and permutated splitting, and controlled for by Monte Carlo splitting. Hence, based on our theoretical review, Monte Carlo splitting, perhaps stratified by task design, could be considered as most robust against the four confounding effects that we listed.

To examine to what degree interactions between confounding effects and splitting methods affected reliability estimates, we systematically applied each splitting method to four different cognitive task datasets. Datasets were selected from three published studies in the fields of gambling-related cognitive processes (Boffo et al., 2018), ethnic stereotypes (Abacioglu et al., 2019), and executive functioning (Hedge et al., 2018). Each dataset was scored with a different non-linear algorithm, commonly used in that literature. Task datasets were from four different paradigms: Approach Avoidance Task (AAT), GNG, IAT, and SST. We compared reliability estimates between splitting methods and task datasets to assess to what degree confounding effects of time, task design, and non-linear scoring were likely to be present. When a splitting method that was confounded with time, task design, or non-linear scoring yielded a different reliability estimate than a splitting method that controlled for these effects, we considered that evidence for the presence of that confounding effect. Note that our hypotheses are non-directional; we did not expect any reliability estimate to be larger or smaller than another estimate, but only expected differences being present between reliability estimates. Exploratively, we examined the combination of resampled splitting and stratification, as well as the shapes of the distributions of the coefficients from resampled splits.

To the best of our knowledge, this is the first paper that comprehensively reviews splitting methods used in the cognitive task literature. Additionally, this is the first study that systematically applies each method. Results can inform researchers which confounding effects may inflate or deflate reliability estimates.

## Methods

### Datasets and scoring algorithms

Table 1 gives an overview of the cognitive task datasets and associated scoring algorithms that we reanalyzed. In all cases, except for the IAT, the number of participants corresponded to those included in the published study. For the IAT, we only included participants who completed this task. Also, conforming to the improved d-score algorithm, we only included participants for whom 10% or less of their responses had a response time (RT) below 300 ms. The implementations of the scoring algorithms were based on the following sources: double difference of median RTs was based on Heuer et al. (2007); d’ was based on the psycho R-package (Makowski, 2018), correcting for extreme values via the log-linear approach (Hautus, 1995); improved d-score was based on Greenwald et al. (2003); and Stop-Signal Reaction Time integration method was based on an R-script provided by Craig Hedge (Hedge et al., 2018).

**Table 1:** Overview of task datasets and scoring algorithms used in the empirical assessment

Task Dataset	Gambling Approach Avoidance Task (AAT) (Boffo et al., 2018)	Go/No-Go (GNG) (Hedge et al., 2018)	Ethnicity-Valence Implicit Association Task (IAT) (Abacioglu et al., 2019)	Stop Signal Task (SST) (Hedge et al., 2018)
#Participants, #trials	48, 128	47, 600	31, 192	45, 600
Scoring algorithm	Double difference of median RTs for correct responses (Heuer et al., 2007)	d’ (Hautus, 1995; Miller, 1996; Williams & Kaufmann, 2012)	D-score for IATs that require a correct response for continuing to the next trial (Greenwald et al., 2003)	Stop-Signal Reaction Time, integration method (Hedge et al., 2018; Logan, 1981)
Scoring conditions (#trials)	Approach gambling (32), avoid gambling (32), approach neutral (32), avoid neutral (32)	Go (450), no-go (150)	Congruent practice (24), incongruent practice (24), congruent test (72), incongruent test (72)	Go (450), stop (150)
Stimuli (#trials)	32 Stimuli (2 approach, 2 avoid)	5 stimuli (90 go, 30 no-go)	4 categories (2 × 6 practice, 2 × 18 test)	None
Trial sequence	Scoring conditions and stimuli in random order	Stimuli in sequence, scoring conditions random within stimuli	Scoring conditions in sequence, stimuli alternated between target and attribute	Scoring conditions: stop delay based on go performance
Design interactions	None	Stimulus with first-second half	Scoring with first-second half and stimulus with odd-even	Scoring with odd-even

Table 1 also shows task conditions for which summary statistics are calculated as part of the task-scoring algorithm (scoring conditions) and task stimuli. The table lists any scoring conditions or stimuli that interacted with first-second or odd-even splitting at design interactions. There were four such interactions: Stimuli of the GNG interacted with first-second splitting because each of the five stimuli was presented in sequences of 120 trials. For the IAT, scoring conditions interacted with first-second splitting, because practice and test trials were administered in sequence. Also for the IAT, stimuli interacted with odd-even splitting, because target and attribute categories alternated. Scoring conditions of the SST interacted with odd-even splitting, because the task’s tracking procedure incremented or decremented stop-signal delay by 50 ms based on whether the previous response was correct or incorrect, respectively.

### Design

We applied first-second, odd-even, permutated, and Monte Carlo splitting to each task dataset. For permutated and Monte Carlo splits, 10,000 replications were conducted and the resulting coefficients averaged via a simple mean. For first-second, odd-even, and permutated splits, reliability was estimated via Spearman-Brown-adjusted Pearson correlations. For Monte Carlo splits, reliability was estimated via Pearson correlations. While not hypothesized, we found that reliability estimates for the AAT could be negative. Spearman-Brown adjustment disproportionally inflates negative values, so for the AAT, we reported and interpreted split-half correlations instead. As a Monte Carlo equivalent of split-half correlations, we sampled with replacement two parts of a half-length instead of full-length.

Each splitting method was conducted with three levels of stratification: no stratification, stratified by scoring conditions, and stratified by each combination of scoring condition and stimulus. With two exceptions, coefficients could be calculated for each combination of splitting method, stratification level, and task: the SST could not be stratified by scoring and stimuli because there was no variation of stimuli within scoring conditions. D-score could not be calculated for the IAT split first-second without stratification, as this method assigned all practice trials to the first part. Hence, in the second part, no summary statistics could be calculated for two of the scoring conditions. Two other exceptions occurred in tasks that sequentially administered an even number of conditions. In such cases, an odd-even split assigned the same trials to each part as an odd-even split stratified by these conditions did, so both splitting methods yielded equivalent results. This applied to the GNG stratified by scoring and stimuli, and IAT stratified by scoring.

Reliability estimates obtained via single-sample splitting methods were qualified as follows: we conceptualized the empirical distributions of non-stratified permutated coefficients as the universe of all possible ways to split a task into two halves. Hence, the percentile of a single reliability estimate in this distribution represents how high or how low this estimate is compared to any random split-half. Similarly, the difference between the percentiles of two reliability estimates represents how much they differ in magnitude, compared to any random split-half. Reliability estimates obtained via resampled splitting methods were qualified by their 95% highest density interval (Kopp et al., 2021). The difference between two distributions of resampled coefficients was qualified by their degree of disjointedness. Disjointedness was calculated as 1 minus the Bhattacharyya coefficient (Bhattacharyya, 1943) so that 0 represented perfectly overlapping distributions and 1 represented perfectly non-overlapping distributions. To illustrate how the magnitude of disjointedness could be interpreted, consider two normal distributions with equal standard deviations (SDs), but different means. Differences between these two distributions that correspond to a small (0.2), medium (0.5), and large (0.8) Cohen’s *d* correspond to disjointedness values of 0.08, 0.20, and 0.31, respectively (Grice & Barrett, 2014).

### Software

Executing each splitting method for each cognitive task dataset and scoring algorithm was not trivial. Hence, we developed an R package, splithalfr, that expands on the functionality provided by existing R packages, such as multicon (Sherman, 2015), psych (Revelle, 2018), and splithalf (Parsons, 2017, 2021). Unique features of the splithalfr are support of Monte Carlo splitting, researcher-provided stratifications and scoring algorithms, nonparametric bias-corrected and accelerated bootstrap confidence intervals (Efron, 1987; Efron & Narasimhan, 2018), and the option to match trials across participants. Each of the cognitive task datasets reanalyzed in this paper has been included in this R package, together with vignettes that serve as tutorials and as a means to replicate the reanalysis. The splithalfr can be installed via CRAN, while its source code is available at https://github.com/tpronk/splithalfr.

## Results

Table 2 displays coefficients for each splitting method, stratification, and task dataset. Below, we report per task how reliability estimates were affected by hypothesized confounding effects. The pattern of GNG, IAT, and SST reliability estimates was consistent with our model of confounding effects, but the AAT was not. Hence, one sub-section summarizes the results of the GNG, IAT, and SST in unison, followed by a second sub-section that summarizes the results of the AAT. Based on our findings in the previous two sub-sections, we examined the stability of reliability estimates with decreased numbers of participants and trials in a third sub-section. In a fourth sub-section, we examine the distributions of reliability estimates of resampled splits and how resampled splits were affected by stratification.

**Table 2:** Coefficients per splitting method, stratification level, and task dataset

Method	Stratification	AAT		GNG		IAT		SST	
		%	Coef	%	Coef	%	Coef	%	Coef
First-second	None	94.20	0.20	0.00	0.84			0.29	0.74
First-second	Scoring	99.67	0.40	0.00	0.84	0.58	0.68	0.25	0.74
First-second	Scoring and Stimuli	89.94	0.15	17.69	0.91	6.02	0.75		
Odd-even	None	23.90	-0.24	73.00	0.94	11.54	0.76	18.71	0.88
Odd-even	Scoring	54.19	-0.08	59.71	0.93	--	--	99.37	0.96
Odd-even	Scoring and Stimuli	89.94	0.15	--	--	95.01	0.89		
Permutated	None		-0.10		0.92		0.82		0.90
		*[-0.45, 0.27]*		*[0.89, 0.96]*		*[0.73, 0.90]*		*[0.83, 0.96]*	
Permutated	Scoring	-0.11 *[-0.46, 0.26]*		0.93 *[0.89, 0.96]*		0.83 *[0.74, 0.91]*		0.90 *[0.83, 0.96]*	
Permutated	Scoring and Stimuli	-0.12 *[-0.49, 0.24]*		0.93 *[0.89, 0.96]*		0.83 *[0.75, 0.91]*			
Monte Carlo	None	0.20 *[-0.17, 0.57]*		0.93 *[0.90, 0.96]*		0.86 *[0.78, 0.93]*		0.91 *[0.84, 0.96]*	
Monte Carlo	Scoring	0.21 *[-0.17, 0.58]*		0.93 *[0.90, 0.96]*		0.86 *[0.79, 0.93]*		0.91 *[0.84, 0.96]*	
Monte Carlo	Scoring and Stimuli	0.35 *[-0.03, 0.70]*		0.93 *[0.90, 0.96]*		0.88 *[0.81, 0.94]*			

*AAT* Approach Avoidance Task, *GNG* Go/No-Go, *IAT* Implicit Association Task, *SST* Stop Signal Task

For the AAT, split-half Pearson correlations are shown, while for the other tasks, split-half Spearman-Brown-adjusted Pearson correlations are shown. The column Coef contains the value of the coefficient, while the column % shows the percentile of this value in the cumulative empirical distribution of non-stratified permutated coefficients. Below each coefficient obtained via resampled splitting, the 95% HDI is shown in italics. Coefficients that could not be calculated are left empty, while coefficients of splitting methods that are equivalent to the splitting method above them are indicated by dashed lines (--)

### GNG, IAT, and SST reliability estimates

Examining confounds with time, reliability estimates of GNG, IAT, and SST, split first-second without stratification fell in the lowest 0.58%. Stratified first-second splits yielded higher estimates for GNG (increasing from 0.84 to 0.91) and IAT (increasing from 0.68 to 0.75), though none exceeded 17.69%. In contrast, estimates from odd-even splits were at least 11.54% without stratification to at least 59.71% with stratification. Hence, confounds with time yielded comparatively low reliability estimates of first-second splitting and this confound could be controlled for via odd-even splitting.

Secondly, we examined confounds with task design. Each case where there was a confound with task design and a single-sample splitting method, we stratified that splitting method so that the confound was controlled for increased reliability estimates. This was the case for GNG split first-second stratified by scoring and stimuli, IAT split odd-even stratified by scoring and stimuli, and SST split odd-even stratified by scoring. In terms of coefficient values, stratification increased estimates by 0.07 to 0.13. For the IAT and SST, stratification increased the reliability estimate by 83.47 and 80.66 percentile points, respectively. For the GNG, stratification increased the reliability estimate by only 17.69 percentile points. The latter increase was low percentile-wise, but high in terms of coefficient values because the non-stratified estimate obtained via first-second splitting was an extremely low outlier (0.00%). Hence, confounds between splitting method and task design yielded low reliability estimates and this confound could be controlled for via stratification.

Thirdly, we examined confounds with non-linear scoring. For the GNG and SST, Monte Carlo estimates were at most 0.01 higher than permutated estimates, which corresponded with the distributions of coefficients being disjoint by at most 17% and 8%, respectively. For the IAT, Monte Carlo estimates were 0.04 to 0.05 higher, which corresponded with a disjointedness of 35–47%. Hence, controlling for confounds of non-linear scoring via Monte-Carlo splitting increased reliability estimates, but only substantially so for the IAT.

Finally, we examined the combination of resampling and stratification. Stratified coefficients were higher than non-stratified coefficients. For the GNG and SST, this difference was negligible, being at most 0.01, which corresponded with a disjointedness of at most 5% for the GNG and 1% for the SST. For the IAT, the difference was slightly larger. Stratification could increase permutated coefficients by 0.01, which corresponded to 10% disjointedness. When split Monte Carlo, reliability estimates stratified by scoring and stimuli were 0.02 higher than non-stratified estimates (corresponding to 20% disjointedness), as well as 0.02 higher than estimates stratified by scoring conditions (corresponding to 15% disjointedness). For the IAT, both for permutated and Monte Carlo coefficients, higher estimates of stratified splits corresponded with slightly narrower HDIs. Hence, across the GNG, SST, and IAT, differences between stratifications of resampled coefficients were modest at most.

### AAT reliability estimates

For the AAT, time interacted with splitting method, but this affected coefficients differently than expected. First-second splitting yielded relatively high correlations, all of which were 89.94% or higher. In contrast, odd-even correlations were lower, even reaching negative values. Hence, confounds with time appeared to have yielded high reliability estimates instead of low estimates.

Secondly, we examined confounds with task design. AAT scoring conditions and stimuli were administered in a random order, so no confounds with any splitting method and task design were expected. Nevertheless, stratifications affected reliability estimates in different ways depending on the splitting method. When split first-second, stratification by scoring increased split-half correlations, but stratification by scoring and stimuli decreased them. When split odd-even, stratification increased split-half correlations, ranging from negative without stratification to positive with stratification by scoring and stimuli. Hence, we conclude that some confound with task design may have been present, but was not well controlled for by our stratification approach.

Thirdly, we examined confounds with non-linear scoring. Permutated coefficients were negative while Monte Carlo coefficients were positive. Hence, non-linear scoring decreased reliability estimates.

Finally, we examined the combination of resampling and stratification. With permutated splits, stratification increased coefficients by 0.02 at most, corresponding to a disjointedness of 4%. With Monte Carlo splits, stratification increased coefficients by 0.15 at most, corresponding to a disjointedness of 31%.

### Distributions of resampled coefficients and means of stratified resampled coefficients

The shapes of distributions of resampled coefficients varied between task datasets. Within tasks datasets, shapes were similar between permutated and Monte Carlo splitting, as well as between stratifications.

**Fig. 1:**
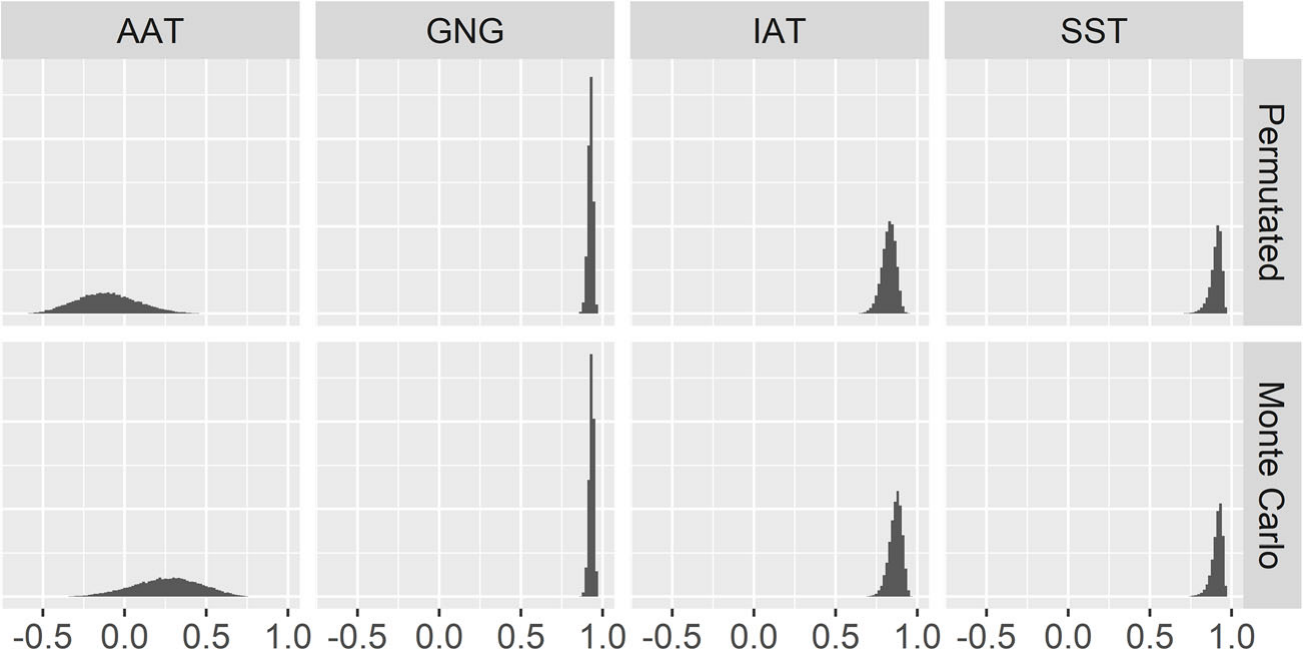
Histograms of coefficients calculated via permutated and Monte Carlo splitting without stratification. For the Approach Avoidance Task (AAT), coefficients are Pearson correlations, while for the Go/No-Go (GNG), Implicit Association Task (IAT), and Stop Signal Task (SST) they are Spearman-Brown*-*adjusted Pearson correlations

For the AAT, GNG, and IAT, distributions were approximately Gaussian. For these task datasets, across permutated and Monte Carlo splitting, combined with any stratification, skewness ranged from -0.71 to -0.17, while excess kurtosis ranged from -0.34 to 0.79. For the SST, distributions were left-skewed (skewness ≤ -1.43) and leptokurtic (excess kurtosis ≥ 3.33). SDs were 0.19 to 0.2 for the AAT, 0.04 to 0.05 for the IAT and SST, and 0.01 to 0.02 for the GNG. Figure 1 shows the distributions of non-stratified coefficients per resampling method and task dataset.

## Discussion

We reviewed four methods for splitting data of cognitive tasks into two parts. Two methods yield a single sample of parts by distributing trials based on their position in sequence administered to the participant (first-second and odd-even). Two methods resample parts by randomly drawing without replacement (permutated) and with replacement (Monte Carlo). Each of these methods can be stratified by task design so that conditions and stimuli are balanced between parts. We reviewed four effects that may confound with splitting methods, thereby affecting the accuracy of split-half reliability estimates. These confounding effects are time, task design, trial sampling, and non-linear scoring.

We systematically applied each single-sample and resample splitting method with different levels of stratification to four cognitive task datasets. Each task had a different design and non-linear-scoring algorithm. These tasks were an AAT scored via double difference of medians; a GNG scored via d’, an IAT scored via d-score, and an SST scored via SSRT integration method. We interpreted differences between reliability estimates in terms of which effects were likely confounding with a splitting method. Exploratively, we examined distributions of resampled estimates.

The pattern of reliability estimates of the GNG, IAT, and SST could be interpreted in terms of hypothesized confounds. These findings are discussed next in unison. We follow with a discussion of the AAT dataset, which had a pattern of results that was more difficult to interpret. However, AAT results could provide indicators for situations in which our model of confounding effects was not applicable. Finally, we propose some future avenues of research into the estimation of the reliability of cognitive task datasets.

For the GNG, IAT, and SST we found confounds between time and first-second splitting. In line with Green et al. (2016), Kopp et al. (2021), Webb et al. (2006), and Williams and Kaufmann (2012), this confound resulted in low reliability estimates for all three tasks. Odd-even splitting controlled for time, resulting in high estimates. For all three tasks, we also found confounds between task design and single-sample splitting methods. Stratifications that controlled for task design resulted in higher estimates. Our findings on task design are in line with the findings of Green et al. (2016), but go against the explanation put forth by Hedge et al. (2018). The latter authors explained high reliability estimates found with the SST as an artifact of the task’s tracking procedure. Our findings indicate that such an artifact resulted in low reliability estimates because stratifying odd-even splits by tracking conditions yielded higher estimates. We found confounds with non-linear scoring, with Monte Carlo estimates being higher than permutated estimates. Only for the IAT was the latter difference substantial.

Exploratively, we examined the combination of stratification and resampling. Stratifying resampled splits yielded slightly higher estimates, most notably for the IAT, split Monte Carlo, stratified by scoring conditions and stimuli. Higher estimates from stratified resampled splits corresponded with a slightly narrower HDI. These findings could imply that resampled splits can be made more equivalent by stratifying them both by task design elements that are part of a task’s scoring algorithm and task design elements that are counterbalanced. However, given that we only found such differences in a limited number of cases, and differences were relatively small, our empirical evidence for the benefits of stratifying resampled coefficients is modest at best.

For the AAT, some split-half correlations were negative. Negative reliabilities can be an artifact due to high correlations between scoring conditions, which could be expected to be averaged out to zero across samples (Parsons et al., 2019). In our results, they did not, even though our resampling procedure had a relatively large number of replications (10,000). Alternatively, the negative correlations may have been caused by multiple negatively correlated dimensions underlying task scores (Cronbach & Hartmann, 1946). As Spearman-Brown adjustment inflates negative correlations, we analyzed correlations instead.

Compared to the GNG and SST, the AAT and IAT showed larger variability within and between splitting methods. This may in part be explained due to the AAT having a relatively small number of trials and the IAT having a relatively small number of participants. A method that could be used to examine such explanations more closely is used in the supplementary materials of Hedge et al. (2018). They examined the reliability of artificially shortened tasks constructed by subsampling trials (i.e., sampling without replacement). In a similar vein, smaller samples could be constructed by subsampling participants. While beyond the scope of this paper, we hope to facilitate such examinations, by offering both subsampling methods in our compendium R-package.

An explanation for the particularly unstable reliability estimates of the AAT could be that it was an irrelevant-feature task. Note that the AAT can be used both in a relevant-feature version and in an irrelevant feature version. In a relevant-feature version, participants are explicitly instructed to approach the focal category in one block and to avoid it in another block (here: approach gambling, avoid other stimuli and avoid gambling, approach other stimuli), structurally similar to the IAT. Here an irrelevant-feature version was used: participants have to make their decision to approach or avoid the stimulus on a characteristic unrelated to the contents of the picture (e.g., approach pictures tilted to the left, avoid pictures tilted to the right). While irrelevant feature versions have their advantages (they are more implicit because there is no explicit instruction relating to the category of interest and the assessment task can be modified into a modification task), the reliability is lower for irrelevant feature versions compared with relevant feature versions (e.g., Field et al., 2011).

The pattern of split-half correlations of the AAT across splitting methods was not consistent with our model of confounding effects for both time and task design. We consider this evidence against the generality of our model of confounding effects. Hence, for the AAT, we do not consider any of the splitting methods that we examined as robust. We do recommend comparing the consistency across splitting methods for diagnosing whether there may be an issue with split-half reliability estimation. Based on our AAT results, we propose the following indicators: negative reliability estimates, exceptionally high estimates with first-second splitting, low estimates with odd-even splitting, finer stratifications not consistently increasing estimates, and estimates of Monte Carlo splitting showing a large difference with estimates from permutated splitting. When assessing a negative reliability estimate, we endorse Parsons and colleagues’ (Parsons et al., 2019) recommendation to treat them as zero, and so essentially consider the corresponding measurement as unsuitable for measuring individual differences.

Exploratively, we examined the distributions of resampled coefficients. Williams and Kaufmann (2012) suggested that wide and platykurtic distributions may indicate issues with trial quality. Based on the findings above, we consider the AAT the only task dataset that had issues with trial quality. In line with Williams and Kaufmann's (2012) suggestions, the range of resampled coefficients for the AAT was relatively high. In contrast with Williams and Kaufmann's (2012) suggestions, the distribution of the AAT was not platykurtic. For the SST, which we deem did not have issues with trial quality, the distribution of resampled coefficients was narrow, skewed to the left, and leptokurtic. Note that the skewness may well have been a ceiling effect due to the reliability estimates of the SST being close to 1. Hence, we found evidence for Williams and Kaufmann’s (2012) suggestion that wide distributions of resampled coefficients may indicate issues with trial quality, but no evidence that the same held for platykurtic distributions. Alternatively, a different method of averaging than a simple mean may be more suitable for averaging resampled reliability estimates that have a non-Gaussian distribution, such as a Fisher z-transformation (Waechter & Stolz, 2015) or averaging via the median (Kopp et al., 2021). For an overview of different averaging methods that may be suitable, see Feldt and Charter (2006).

In summary, we found strong evidence for effects of time and task design, which yielded relatively low reliability estimates when confounded with a splitting method. We found weak evidence for confounds with non-linear effects, which could be controlled for via Monte Carlo splitting. Coefficients of resampled splits were only modestly affected by stratification. Based on our theoretical model, we have recommended stratified Monte Carlo splitting as being most robust against the confounding effects that we hypothesized. Based on the confounding effects for which we found evidence in our empirical analysis, we add that in most of the datasets studied here, resampled splitting via permutation or resampled splitting without stratification were similarly robust. We also found evidence that our model of confounding effects did not apply to all task datasets, in which case we do not endorse any splitting method. We listed some indicators for when a dataset may not meet model assumptions.

To the best of our knowledge, this is the first comprehensive review and empirical study of splitting methods that have been used for cognitive tasks. Given the focus of this paper, a range of other aspects that are relevant to reliability estimation were not covered. Below, we list four aspects and make some suggestions on how to proceed.

Firstly, reliability is not only a function of task and population sample but also of scoring algorithms. In the present study, each task dataset was scored with a different scoring algorithm, selected for having been applied to their corresponding task in the literature. Within a task and sample, the methods we outlined could be used to compare scoring algorithms, similar to Glashouwer et al. (2013). Secondly, we used Spearman-Brown-adjusted Pearson correlations for being the most common reliability coefficient in the cognitive task literature. Alternative coefficients may be useful in particular cases. For instance, alpha could be applied in cases where splitting into more than two parts is desired (Green et al., 2016) and Angoff-Feldt when the number of trials varies between each part (Feldt & Charter, 2003; Walker, 2005). An Intraclass Correlation Coefficient (ICC) could be calculated for assessing consistency and absolute agreement between scores (Koo & Li, 2016; Shrout & Fleiss, 1979). A third aspect is that we did not explicitly review measurement models of reliability coefficients beyond linear and non-linear transformations. One measurement model that is interesting in this light is an application of classical test theory models to reaction time data (Rouder & Haaf, 2019). A fourth aspect concerns methods for assessing the magnitude of reliability estimates and differences between reliability estimates. We applied descriptive statistics of percentages, where single-sample reliability estimates were assessed via their percentiles in permutated distributions and resampled reliability estimates were compared via their degree of disjointedness between empirical distributions. While we deem this method as robust against non-linearity and more comprehensive than a significance test, it might be less informative when comparing relatively high reliability estimates.

In conclusion, we return to the issue prompting our examination of methods for estimating reliability, which was the low reliability with which individual differences could be measured by several cognitive tasks (Hedge et al., 2018). The primary method for assessing reliability used by Hedge and colleagues was test-retest reliability. We focused on split-half reliabilities for their suitability in cases where multiple administrations of a task are not feasible or a score may vary over test-retest timeframes. In our empirical analyses, we examined whether splitting methods that were more robust against hypothesized confounding effects would yield different reliability estimates. For cases where our model of confounding effects applied, more robust splitting methods yielded higher reliability estimates than less robust splitting methods. Reliabilities estimated via splitting methods we deemed as most robust were strikingly high, exceeding 0.82 for the IAT and 0.90 for the GNG and SST. Hence, we conclude that for the datasets included in our reanalysis, cognitive tasks may well have been able to measure individual differences, but that these differences may be relatively unstable over time (Kopp et al., 2021). In practice, this may make cognitive tasks suitable for cross-sectional research of individual differences, but not for longitudinal research. A more thorough assessment could involve replicating this finding across a larger number of task datasets. In support of such a venture, all splitting methods described in this paper are available in the R package that serves as a compendium to this paper.

## References

[CR1] Abacioglu CS, Zee M, Hanna F, Soeterik IM, Fischer AH, Volman M (2019). Practice what you preach: The moderating role of teacher attitudes on the relationship between prejudice reduction and student engagement. Teaching and Teacher Education.

[CR2] Bhattacharyya A (1943). On a measure of divergence between two statistical populations defined by their probability distributions. Bulletin of the Calcutta Mathematical Society.

[CR3] Boffo M, Smits R, Salmon JP, Cowie ME, de Jong DTHA, Salemink E, Collins P, Stewart SH, Wiers RW (2018). Luck, come here! Automatic approach tendencies toward gambling cues in moderate- to high-risk gamblers. Addiction.

[CR4] Chapman A, Devue C, Grimshaw GM (2019). Fleeting reliability in the dot-probe task. Psychological Research.

[CR5] Cooper SR, Gonthier C, Barch DM, Braver TS (2017). The role of psychometrics in individual differences research in cognition: A case study of the AX-CPT. Frontiers in Psychology.

[CR6] Cortina JM (1993). What Is Coefficient Alpha ? An Examination of Theory and Applications. Journal of Applied Psychology.

[CR7] Cronbach LJ, Hartmann W (1946). A note on negative reliabilities. Educational and Psychological Measurement.

[CR8] de Hullu E, de Jong PJ, Sportel BE, Nauta MH (2011). Threat-related automatic associations in socially anxious adolescents. Behaviour Research and Therapy.

[CR9] de Vet HCW, Mokkink LB, Mosmuller DG, Terwee CB (2017). Spearman–Brown prophecy formula and Cronbach’s alpha: different faces of reliability and opportunities for new applications. Journal of Clinical Epidemiology.

[CR10] Efron B (1987). Better Bootstrap Confidence Intervals. Journal of the American Statistical Association.

[CR11] Efron, B., & Narasimhan, B. (2018). *bcaboot: Bias Corrected Bootstrap Confidence Intervals*.

[CR12] Enock PM, Hofmann SG, McNally RJ (2014). Attention bias modification training via smartphone to reduce social anxiety: A randomized, controlled multi-session experiment. Cognitive Therapy and Research.

[CR13] Feldt LS, Charter RA (2003). Estimating the Reliability of a Test Split into Two Parts of Equal or Unequal Length. Psychological Methods.

[CR14] Feldt LS, Charter RA (2006). Averaging internal consistency reliability coefficients. Educational and Psychological Measurement.

[CR15] Field M, Caren R, Fernie G, De Houwer J (2011). Alcohol approach tendencies in heavy drinkers: comparison of effects in a Relevant Stimulus-Response Compatibility task and an approach/avoidance Simon task. Psychology of Addictive Behaviors.

[CR16] Glashouwer K a, Smulders FTY, De Jong PJ, Roefs A, Wiers RW (2013). Measuring automatic associations: Validation of algorithms for the Implicit Association Test (IAT) in a laboratory setting. Journal of Behavior Therapy and Experimental Psychiatry.

[CR17] Green SB, Yang Y, Alt M, Brinkley S, Gray S, Hogan T, Cowan N (2016). Use of Internal Consistency Coefficients for Estimating Reliability of Experimental Tasks Scores. Psychonomic Bulletin & Review.

[CR18] Greenwald AG, McGhee DE, Schwartz JL (1998). Measuring individual differences in implicit cognition: the implicit association test. Journal of Personality and Social Psychology.

[CR19] Greenwald AG, Nosek BA, Banaji MR (2003). Understanding and using the Implicit Association Test: I. An improved scoring algorithm. Journal of Personality and Social Psychology.

[CR20] Grice JW, Barrett PT (2014). A Note on Cohen’s Overlapping Proportions of Normal Distributions. Psychological Reports.

[CR21] Hautus MJ (1995). Corrections for extreme proportions and their biasing effects on estimated values of d′. Behavior Research Methods, Instruments, & Computers.

[CR22] Hedge C, Powell G, Sumner P (2018). The reliability paradox: Why robust cognitive tasks do not produce reliable individual differences. Behavior Research Methods.

[CR23] Heuer K, Rinck M, Becker ES (2007). Avoidance of emotional facial expressions in social anxiety: The Approach-Avoidance Task. Behaviour Research and Therapy.

[CR24] Koo TK, Li MY (2016). A Guideline of Selecting and Reporting Intraclass Correlation Coefficients for Reliability Research. Journal of Chiropractic Medicine.

[CR25] Kopp, B., Lange, F., & Steinke, A. (2021). *The Reliability of the Wisconsin Card Sorting Test in Clinical Practice*. 10.1177/1073191119866257 10.1177/1073191119866257 PMC7780274 31375035

[CR26] Lancee J, Yasiney SL, Brendel RS, Boffo M, Clarke PJF, Salemink E (2017). Attentional bias modification training for insomnia: A double-blind placebo controlled randomized trial. PLoS ONE.

[CR27] Logan, G. D. (1981). Attention, automaticity, and the ability to stop a speeded choice response. *Attention and Performance IX*, 205–222.

[CR28] MacLeod JW, Lawrence MA, McConnell MM, Eskes GA, Klein RM, Shore DI (2010). Appraising the ANT: Psychometric and Theoretical Considerations of the Attention Network Test. Neuropsychology.

[CR29] Makowski D (2018). The Psycho Package: An Efficient and Publishing-Oriented Workflow for Psychological Science. Journal of Open Source Software.

[CR30] Miller J (1996). The sampling distribution of d’. Perception & Psychophysics.

[CR31] Novick MR, Lewis C (1967). Coefficient Alpha and the Reliability of Composite Measurements. Psychometrika.

[CR32] Parsons, S. (2017). *splithalf: Calculate Task Split Half Reliability Estimates*. 10.6084/m9.figshare.5559175.v2

[CR33] Parsons S (2021). Splithalf: Robust estimates of split half reliability. Journal of Open Source Software.

[CR34] Parsons, S., Kruijt, A.-W., & Fox, E. (2019). Psychological Science Needs a Standard Practice of Reporting the Reliability of Cognitive-Behavioral Measurements. *Advances in Methods and Practices in Psychological Science*, 1–18. 10.1177/2515245919879695

[CR35] Revelle, W. (2018). psych: Procedures for Personality and Psychological Research. https://cran.r-project.org/package=psych

[CR36] Rouder JN, Haaf JM (2019). A psychometrics of individual differences in experimental tasks. Psychonomic Bulletin and Review.

[CR37] Schmitz EA, Jansen BRJ, Wiers RW, Salemink E (2019). Do implicitly measured math–anxiety associations play a role in math behavior?. Journal of Experimental Child Psychology.

[CR38] Sherman, R. A. (2015). multicon: Multivariate Constructs. R package version 1.6. https://cran.r-project.org/package=multicon

[CR39] Shrout PE, Fleiss JL (1979). Intraclass Correlations : Uses in Assessing Rater Reliability. Psychological Bulletin.

[CR40] Waechter S, Stolz JA (2015). Trait Anxiety, State Anxiety, and Attentional Bias to Threat: Assessing the Psychometric Properties of Response Time Measures. Cognitive Therapy and Research.

[CR41] Waechter S, Nelson AL, Wright C, Hyatt A, Oakman J (2014). Measuring attentional bias to threat: Reliability of dot probe and eye movement indices. Cognitive Therapy and Research.

[CR42] Walker DA (2005). A Comparison of the Spearman-Brown and Flanagan-Rulon Formulas for Split Half Reliability under Various Variance Parameter Conditions. Journal of Modern Applied Statistical Methods.

[CR43] Warrens, M. J. (2015). On Cronbach’s Alpha as the Mean of All Split-Half Reliabilities. In *Quantitative Psychology Research* (Vol. 140, Issue August, pp. 292–300). Springer International Publishing. 10.1007/978-3-319-19977-1

[CR44] Warrens MJ (2017). Transforming intraclass correlation coefficients with the Spearman–Brown formula. Journal of Clinical Epidemiology.

[CR45] Webb, N. M., Shavelson, R. J., & Haertel, E. H. (2006). Reliability Coefficients and Generalizability Theory. In C. Rao & S. Sinharay (Eds.), Handbook of Statistics (Vol. 26, pp. 81–124). Elsevier. 10.1016/S0169-7161(06)26004-8

[CR46] Williams BJ, Kaufmann LM (2012). Reliability of the Go/No Go Association Task. Journal of Experimental Social Psychology.

